# Paliperidone palmitate in non-acute patients with schizophrenia previously unsuccessfully treated with risperidone long-acting therapy or frequently used conventional depot antipsychotics

**DOI:** 10.1177/0269881115586284

**Published:** 2015-08

**Authors:** A Schreiner, P Bergmans, P Cherubin, S Keim, P-M Llorca, B Cosar, A Petralia, G Corrivetti, L Hargarter

**Affiliations:** 1 EMEA Medical Affairs, Janssen Cilag GmbH, Neuss, Germany; 2 Biometrics & Reporting, Janssen Cilag Benelux, Tilburg, the Netherlands; 3 EMEA Medical Affairs, Janssen Cilag, Issy-les-Moulineaux, France; 4 Global Clinical Operations EMEA Medical Affairs, Janssen Cilag, Barcarena, Portugal; 5 CHRU Clermont-Ferrand, Hôpital Gabriel Montpied, Clermont-Ferrand, France; 6 Gazi University Medical Faculty, Ankara, Turkey; 7 UOPI of Psychiatry, AOU Policlinico Vittorio Emanuele, Catania, Italy; 8 UOSM Distretto D, Pontecagnano-Faiano (Sa), Italy

**Keywords:** Non-acute, paliperidone palmitate, schizophrenia, switching, injectable antipsychotic

## Abstract

PALMFlexS, a prospective multicentre, open-label, 6-month, phase IIIb interventional study, explored tolerability, safety and treatment response in adults (*n* = 231) with non-acute but symptomatic schizophrenia switching to flexibly dosed paliperidone palmitate (PP) after unsuccessful treatment with risperidone long-acting injectable therapy (RLAT) or conventional depot antipsychotics (APs). Treatment response was measured by change in Positive and Negative Syndrome Scale (PANSS) total score from baseline (BL) to last-observation-carried-forward (LOCF) endpoint (EP). Safety and tolerability assessments included Extrapyramidal Symptom Rating Scale (ESRS) total score and treatment-emergent adverse events. Significant reductions in mean PANSS total score were observed for all groups (−7.5 to −10.6; *p* ⩽ 0.01 [BL to LOCF EP]). After switching to PP, more than 50% of all patients achieved ⩾20% and one-third of RLAT-treated patients even achieved ⩾50% improvement in PANSS total score. Across groups, there were significant improvements (*p* < 0.05) in symptom severity as measured by Clinical Global Impression-Severity (CGI-S; trend for improvement with RLAT; *p* = 0.0568), subjective well-being, medication satisfaction, and patient functioning with PP. PP was generally well tolerated. Clinically relevant benefits were observed in non-acute patients with schizophrenia switched from RLAT or conventional depot APs to PP.

## Introduction

Despite some level of symptom stabilization following treatment for acute exacerbations of schizophrenia, maintenance treatment is critical for non-acute patients to prevent relapse and hospitalization, and ensure optimal long-term outcomes (National Institute for Health and Care Excellence, 2014), including improved psychosocial functioning and overall quality of life (Leucht et al., 2012).

Current guidelines for the maintenance treatment of schizophrenia include the use of oral antipsychotics (APs) and long-acting injectable antipsychotic therapies (LATs) (National Institute for Health and Care Excellence, 2014). Use of LATs has been associated with a significantly lower risk of relapse compared with oral AP formulations (Leucht et al., 2011), although data from some recent randomized controlled trials (RCTs) and meta-analyses are ambiguous (Kishimoto et al., 2013, 2014), with results depending on variables such as study design and methodology (Kirson et al., 2013). In some studies that have compared conventional depot APs and atypical LATs, it was found, for instance, that patients previously treated with conventional depot APs could be safely and effectively switched to risperidone long-acting injectable therapy (RLAT) with a reduction in the severity of movement disorders compared with their previous treatment (Turner et al., 2004). Also, treatment with RLAT was shown to be more effective in improving symptoms of schizophrenia and substance abuse (Rubio et al., 2006) and led to lower rates of hospitalization compared with conventional depot APs (Grimaldi-Bensouda et al., 2012).

Paliperidone palmitate (PP) is an atypical LAT designed for once-monthly intramuscular (IM) injection (Hough et al., 2009) that is indicated in the EU for maintenance treatment of adult patients with schizophrenia previously stabilized with paliperidone or risperidone (Xeplion SmPC, 2015). PP has been developed as an aqueous suspension. Due to its low solubility, dissolution is slow, thereby enabling hydrolysis and delivery of the active drug, paliperidone, into the systemic circulation (Gopal et al., 2010; Samtani et al., 2009). An initiation regimen (150 mg eq. [Day 1]/ 100 mg eq. [Day 8], both by IM injection in the deltoid) is recommended for patients switching from oral APs to PP to enable early achievement of therapeutic plasma concentrations, followed thereafter by once-monthly injections within the available dose range (Xeplion SmPC, 2015). In patients switching from conventional depot APs or RLAT, PP should be initiated using an appropriate maintenance dose at the next scheduled injection, and monthly thereafter.

A recent 18-month study in patients with schizophrenia demonstrated significant improvements in outcomes such as remission rates and functioning with PP compared with conventional depot APs (Romero Guillena et al., 2014). A year-long naturalistic study showed that PP was well tolerated (Attard et al., 2014) and resulted in a significant decrease in the number of hospital admissions and the mean number of bed days per patient per year compared with previous AP treatments (Taylor and Olofinjana, 2014). Furthermore, a review of randomized, placebo-controlled trials of conventional depot APs (haloperidol decanoate [Hal-Dec], bromperidol decanoate, and fluphenazine decanoate [Flu-Dec]) and PP, to compare the benefit–risk ratio in patients with schizophrenia, demonstrated that the numbers-needed-to-treat (i.e. number of patients who will likely need to be treated to benefit a single patient) were similar to the conventional depot APs assessed, but the numbers-needed-to-harm (i.e. number of patients who will likely need to be treated to cause a single patient to experience harm) favoured PP over conventional depot APs assessed (Gopal et al., 2011).

The primary objective of this analysis was to explore tolerability, safety and treatment response of switching to flexible doses of PP in a group of adults with non-acute but symptomatic schizophrenia following unsuccessful treatment with either RLAT or conventional depot APs. This is the first study exploring patients who switch from either RLAT or conventional depot APs to flexibly dosed PP. In the RCTs of PP used for regulatory purposes, patients previously treated with RLAT or a conventional depot AP were excluded. In addition, in this pragmatic study patients had higher rates of comorbidities, comedications and substance abuse. Secondary objectives were to explore how treatment outcomes may guide recommendations for use of, and transition to, PP in patients with schizophrenia, including clinically relevant information on maintenance dosing.

## Materials and methods

### Study design

The Paliperidone Palmitate Flexible Dosing in Schizophrenia (PALMFlexS) trial was a prospective non-randomized, single-arm, multicentre, open-label, 6-month phase IIIb interventional study in patients with schizophrenia previously unsuccessfully treated with either an oral AP, RLAT or conventional depot AP (Clinical trials.gov number: NCT01281527). A total of 160 sites in 21 countries took part in the study (see Appendix 1, supplementary materials). Prior to trial initiation, the protocol was reviewed and approved by an independent ethics committee in all participating countries. The trial was performed in accordance with the Declaration of Helsinki. Trial patients were informed of the risks and benefits of the trial. Written informed consent was obtained from each patient before any trial-related activities.

The study consisted of a 7-day screening period, a 6-month study period, and an optional extension phase. This manuscript reports results from the 6-month study period. The screening period included a 2-day oral tolerability test with paliperidone extended-release (ER) tablets for patients without source documentation of previous risperidone or paliperidone exposure. Only patients demonstrating an ability to tolerate the drug, as judged by the treating physician, were eligible to enter the 6-month study period. The start of the 6-month study period was defined as the day of the first PP injection.

### Patients

Eligible participants were males and females aged ⩾18 years with a DSM-IV diagnosis of schizophrenia, being non-acute but symptomatic. Patients had to be on an adequate therapeutic dose of another LAT and clinically stable (change in Clinical Global Impression-Severity [CGI-S] ⩽1) in the 4 weeks before enrolment, and had previously been treated with either RLAT or one of the following conventional depot APs: Hal-Dec, flupentixol decanoate [Fpt-Dec], Flu-Dec, or zuclopenthixol decanoate [Zuc-Dec]. A patient’s previous treatment was considered unsuccessful due to one or more of the following criteria: lack of efficacy (baseline [BL] Positive and Negative Syndrome Scale [PANSS] total score ⩾70 or ⩾2 items scoring ⩾4 in the PANSS positive or negative subscale or ⩾3 items scoring ⩾4 in the PANSS general psychopathology subscale), lack of tolerability or safety (the presence of clinically relevant side effects), lack of compliance, or the patient’s wish. An additional key inclusion criterion was that at the discretion of the investigator the patient may benefit from a switch of AP medication to PP.

A psychiatric diagnosis due to direct pharmacological effects of a substance or a general medical condition led to exclusion from the study, as did a history or current symptoms of tardive dyskinesia or neuroleptic malignant syndrome; pregnancy or breast feeding; and known allergies, hypersensitivity, or intolerance to risperidone or paliperidone or its excipients. Patients who were AP treatment-naive; receiving clozapine during the last 3 months prior to the start of the study; or considered to be at imminent risk of suicide even after clinical intervention were also excluded from the study. Patients with a current substance use or abuse, with the exception of intravenous drug use, were eligible for enrolment and no exclusions were applied based on body mass index (BMI).

### Treatment

No randomization or blinding procedures were performed in this study. The first PP administration was initiated in place of the next scheduled injection of the previous LAT, and was given in the deltoid muscle. The first PP dose was within the range of 50–150 mg eq. depending upon the dose of previous AP and the clinical status of the patient. Subsequently, PP was administered once monthly on Days 31, 61, 91, 121 and 151 (±7 days) using flexible dosages within the range of 50–150 mg eq. based upon the decision of the treating physician. In line with the PP EU label, an injection of PP 8 days after the first PP administration was not recommended (Xeplion SmPC, 2015).

Oral APs and other psychotropic medications that were administered prior to the start of the study for reasons other than the disease itself (e.g. sleep induction or sedation) could be continued during the study at a stable dose. In case of worsening of psychotic symptoms between visits requiring immediate intervention, oral AP medication, preferably paliperidone ER, could be given within the approved dose range. Benzodiazepines that were newly initiated during the study were allowed for rescue medication, preferably not for longer than 10 consecutive days. Benztropine mesylate or biperidene up to 4 mg/day or trihexyphenidyl up to 10 mg/day could be used for the treatment of extrapyramidal motor symptoms (EPMS). The investigators were asked to re-evaluate the need for concomitant APs, benzodiazepines and anticholinergic medication on an ongoing basis.

### Efficacy assessments

The objective of this study was to explore tolerability, safety and treatment response of switching patients with schizophrenia from RLAT or conventional depot AP to PP. PANSS was rated by a trained and qualified investigator. Actual values and changes from BL (Day 1 to last-observation-carried-forward [LOCF] endpoint [EP] at 6 months or early discontinuation) in PANSS total score and CGI-S scale score were recorded as part of the primary efficacy assessments.

Secondary outcomes included relative frequency distributions for CGI-S and Clinical Global Impression-Change (CGI-C) scores; change from BL to LOCF EP for PANSS subscales; PANSS Marder Factor Scores and actual values and changes from BL in the Personal and Social Performance (PSP) total score. The PSP total score was determined from four PSP domain scores of socially useful activities, personal and social relationships, self-care, and disturbing and aggressive behaviour, each rated on a 6-point scale (ranging from 0 [absent] to 5 [very severe]) and converted to one total score ranging from 1 to 100. Three categories of functioning were derived from the PSP total score (functioning so poorly as to require intensive support or supervision [PSP total score <31]; varying degrees of disability [PSP total score 31–70]; mild degree of difficulty [PSP total score 71–100]). Additional secondary outcome measures encompassed the Subjective Well-being under Neuroleptics-Scale (SWN-S) (short form) and Mini International Classification of Functionality, Disability and Health (ICF) Rating for Activity and Participation Disorders in Psychological Illnesses (Mini-ICF-APP) scale (Baron and Linden, 2009; Linden and Baron, 2005; Molodynski et al., 2013). The Mini-ICF-APP scale evaluates patients’ functional abilities and disabilities relevant for aspects of life such as evaluation of early retirement, sheltered jobs and reintegration. Each dimension is rated on a Likert scale ranging from 0 (no disability) to 4 (total disability). Patient satisfaction with their AP treatment was assessed using the 14-item Treatment Satisfaction Questionnaire for Medication (TSQM) and physician treatment satisfaction using a 7-point categorical scale. Sleep and daytime drowsiness were evaluated using an 11-point categorical rating scale.

### Safety and tolerability

EPMS were assessed by Extrapyramidal Symptom Rating Scale (ESRS) total score (Chouinard et al., 1980). Vital sign measurements and body weight were recorded and BMI was calculated. Adverse events (AEs) were reported, either directly by the patient or indirectly obtained by means of interviewing patients at study visits. Treatment-emergent adverse events (TEAEs) were defined as AEs that were new in onset or were aggravated in severity following initiation of PP. All reported AEs were coded using the Medical Dictionary for Regulatory Activities (MedDRA version 13.0). As this was a pragmatic study and data on prolactin plasma levels with PP have been extensively collected during the clinical development programme, no regular lab tests were conducted. However, investigators could measure laboratory values including prolactin at their own discretion.

### Data analysis

The results of this study are summarized descriptively. The number of patients switched from RLAT or conventional depot APs to PP was targeted at approximately 200, based on approximately 40 patients switching from each of the prior LATs. The intent-to-treat (ITT) population comprised all patients who received PP at least once during the course of the study. Change versus BL was tested by means of the Wilcoxon signed-rank test. Tolerability and safety were evaluated throughout the study on the safety ITT population.

## Results

### Demographics and patient disposition

**Figure 1.**
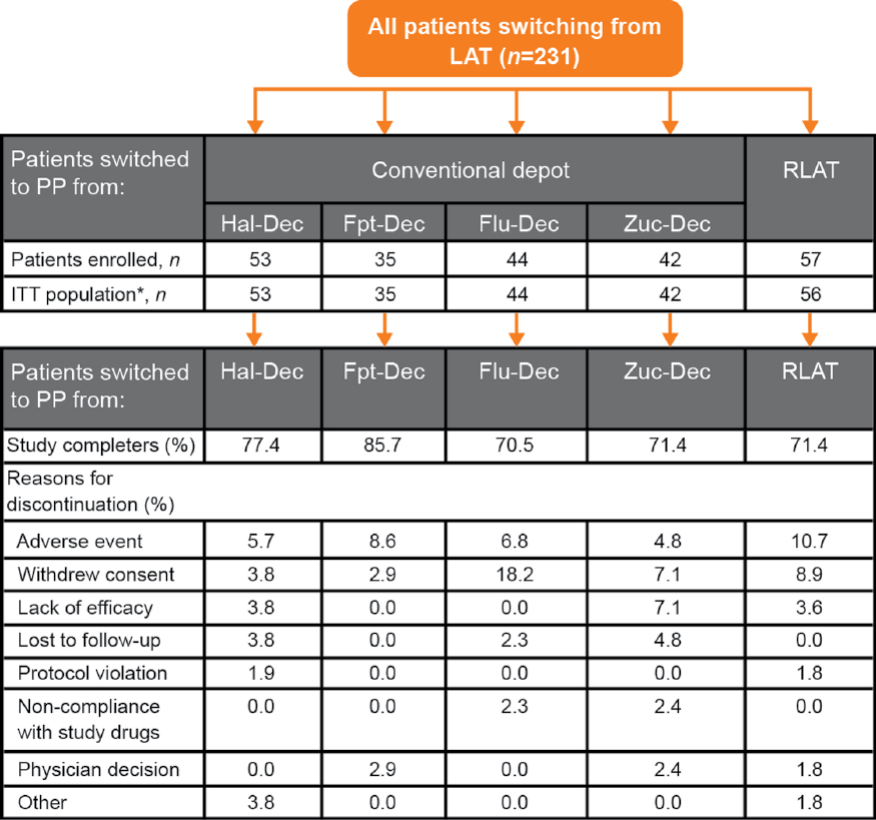
Patient disposition. *Patients who received at least one dose of study drug. Flu-Dec: fluphenazine decanoate; Fpt-Dec: flupentixol decanoate; Hal-Dec: haloperidol decanoate; ITT: intention to treat; LAT: long-acting injectable antipsychotic therapy; PP: paliperidone palmitate; RLAT: risperidone long-acting injectable therapy; Zuc-Dec: zuclopenthixol decanoate.

Patient disposition is described in Figure 1. Overall, 231 patients switched to PP from conventional depot APs (*n* = 174) or RLAT (*n* = 57). There was variation in the reasons for switching across groups, with lack of efficacy ranging from 12.5% for patients switching to PP from RLAT to 54.3% in the Fpt-Dec group, while those switching due to patient’s wish ranged from 67.9% in the RLAT group to 26.2% in the Zuc-Dec group. The proportion of patients completing the study varied from 70.5% in patients switched to PP from Flu-Dec to 85.7% in patients switched from Fpt-Dec. The most common reasons for discontinuation varied according to the LAT from which the patients switched. Discontinuation due to withdrawal of consent varied from 18.2% (8/44) in the group switched to PP from Flu-Dec treatment to 2.9% (1/35) in those switched from Fpt-Dec. In addition, discontinuation due to AEs varied from 10.7% (6/56) in those switched to PP from RLAT to 4.8% (2/42) in those switched from Zuc-Dec.

BL characteristics are summarized in Table 1. Characteristics at BL were comparable across previous treatment groups with the exception of some numerical between-group differences for mean age (standard deviation [SD]) (ranging from 39.9 [11.0] years [RLAT] to 44.4 [9.4] years [Hal-Dec]), mean weight (78.9 [15.4] kg [Hal-Dec] to 87.0 [19.5] kg [Fpt-Dec]), proportion of males (57.1% [Zuc-Dec] to 69.8% [Hal-Dec]), and BL PANSS total scores (67.7 [20.3; RLAT] to 75.7 [13.2; Hal-Dec]). The percentage of patients with a BMI of ⩾25 kg/m^2^ varied from 59.6% (Hal-Dec) to 78.6% (Zuc-Dec).

**Table 1.** BL characteristics of the ITT population.

	Patients switched from:				
	Conventional depot:				RLAT
	Hal-Dec	Fpt-Dec	Flu-Dec	Zuc-Dec	
**ITT population (*n*)**	53	35	44	42	56
**Male, %**	69.8	62.9	63.6	57.1	64.3
**Mean age, years (SD)**	44.4 (9.4)	42.1 (12.7)	44.2 (10.5)	42.1 (10.7)	39.9 (11.0)
**Mean age at diagnosis, years (SD)**	32.8 (8.6)	29.1 (9.7)	31.5 (9.6)	31.1 (11.5)	29.9 (10.1)
**Diagnosis of paranoid schizophrenia, %**	81.1	74.3	81.8	78.6	71.4
Patients with ⩾1 comorbidity, *n* (%)*	29 (54.7)	15 (42.9)	25 (56.8)	25 (59.5)	32 (57.1)
**Mean BL weight, kg (SD)**	78.9 (15.4)	87.0 (19.5)	80.0 (19.4)	86.8 (18.2)	85.2 (19.5)
**Mean BL BMI, kg/m^2^ (SD)**	27.3 (5.9)	28.4 (5.6)	27.5 (5.5)	29.7 (6.4)	28.5 (5.8)
**Mean BL PANSS total score (SD)**	75.7 (13.2)	75.0 (15.9)	75.0 (15.4)	74.8 (16.3)	67.7 (20.3)
**Mean BL CGI-S score (SD)**	4.2 (0.9)	3.9 (0.8)	4.0 (1.0)	4.1 (1.0)	3.7 (1.2)
**Number of previous hospitalizations, %**					
** None**	7.5	11.4	6.8	9.5	12.5
** 1–3**	45.3	45.7	34.1	38.1	51.8
** ⩾4**	47.2	42.9	59.1	52.4	35.7

* Individual patients can be labelled for >1 comorbidity.

BL: baseline; BMI: body mass index; CGI-S: Clinical Global Impression-Severity; ITT: intention to treat; PANSS: Positive and Negative Syndrome Scale; SD: standard deviation.

The proportion of patients who received 150 mg eq. PP as the first dose ranged from 26.8% for patients previously treated with RLAT to 45.5% and 45.3% for patients previously treated with Flu-Dec and Hal-Dec, respectively (Table 2). The mean average dose of PP (SD) after the initial dose regimen ranged between 105.0 (32.6) mg eq. for patients switched to PP from RLAT and 113.9 (30.7) mg eq. for those switched from Zuc-Dec treatment. Across all groups, 40.5–50.0% of patients had no change in dose over the course of the study. The most common reasons for dose adjustments were suboptimal efficacy (dose increases in 25.0% [RLAT] to 35.7% [Zuc-Dec] of patients) and patients responding well to treatment (dose decreases in 9.5% [Zuc-Dec] to 28.6% [Fpt-Dec] of patients).

**Table 2.** PP dosing during the study (ITT population).

	Patients switched from:				
	Conventional depot				RLAT *n* = 56
	Hal-Dec *n* = 53	Fpt-Dec *n* = 35	Flu-Dec *n* = 44	Zuc-Dec *n* = 42	
**Mean dose of previous LAT, mg (SD)**	138.1 (127.0)*	96.9 (119.9)^†^	36.5 (18.9)^‡^	398.5 (353.4)^§^	50.2 (24.7)^¶^
**Mean modal PP main-tenance dose mg eq. (SD)**	111.3 (33.5)	107.6 (33.9)	107.1 (32.3)	112.5 (34.2)	104.2 (34.9)
**First PP dose received, % of patients**					
** 50 mg eq.**	0.0	2.9	11.4	7.1	10.7
** 75 mg eq.**	24.5	25.7	20.5	14.3	16.1
** 100 mg eq.**	30.2	42.9	22.7	38.1	46.4
** 150 mg eq.**	45.3	28.6	45.5	40.5	26.8
**Last PP dose received, % of patients**					
** 50 mg eq.**	5.7	14.3	4.5	7.1	10.7
** 75 mg eq.**	18.9	11.4	20.5	7.1	23.2
** 100 mg eq.**	22.6	31.4	38.6	42.9	30.4
** 150 mg eq.**	52.8	42.9	36.4	42.9	35.7

* 75.5% of patients had a monthly dosing interval (mean monthly dose: 100.9 mg); ^†^54.3% of patients had a monthly dosing interval (mean monthly dose: 36.8 mg); ^‡^65.9% of patients had a monthly dosing interval (mean monthly dose: 28.0 mg); ^§^38.1% of patients had a monthly dosing interval (mean monthly dose: 165.3 mg) and 38.1% had a biweekly dosing interval (mean biweekly dose: 267.5 mg); ^¶^100% of patients had a biweekly dosing interval (mean biweekly dose: 50.2 mg).

PP: paliperidone palmitate; SD: standard deviation.

### Efficacy outcomes

**Figure 2.**
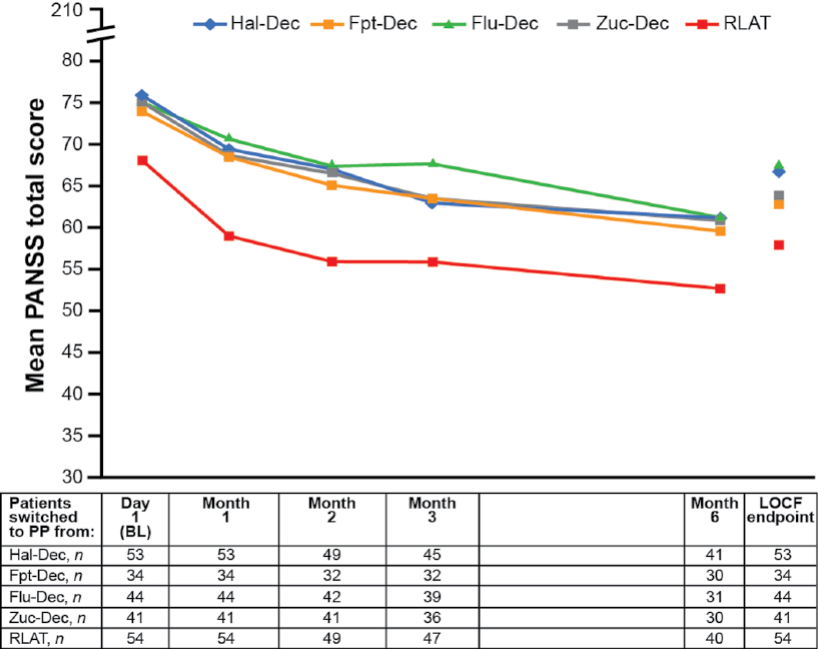
Mean PANSS total score over time (*n* = 226). *p* < 0.0001 vs. BL for all data points except: Month 2, Zuc-Dec: *p* = 0.0003 and LOCF EP, Fpt-Dec: *p* = 0.0006; Flu-Dec: *p* = 0.0029; Zuc-Dec: *p* = 0.0007; RLAT: *p* = 0.0001. Within-group difference was tested using the Wilcoxon signed-rank test. BL: baseline; Flu-Dec: fluphenazine decanoate; Fpt-Dec: flupentixol decanoate; Hal-Dec: haloperidol decanoate; LOCF: last observation carried forward; PANSS: Positive and Negative Syndrome Scale; PP: paliperidone palmitate; RLAT: risperidone long-acting injectable therapy; Zuc-Dec: zuclopenthixol decanoate.

Mean PANSS total score (SD) was reduced significantly from BL to LOCF EP in all groups, ranging from −7.5 (19.4; *p* = 0.0029) in patients switched from Flu-Dec to −10.6 (21.5; *p* = 0.0007) in patients switched from Zuc-Dec (Figure 2, Table 3). Mean PANSS total scores for patients switched from Hal-Dec, Fpt-Dec, Flu-Dec and Zuc-Dec were similar at BL and throughout the study, and were noticeably lower at BL and throughout the study for patients switched from RLAT (Figure 2).

**Table 3.** Efficacy outcomes.*

	Patients switched from:				
	Conventional depot				RLAT
	Hal-Dec	Fpt-Dec	Flu-Dec	Zuc-Dec	
**Mean PANSS total score, *n***	53	34	44	41	54
** BL (SD)**	75.7 (13.2)	73.7 (14.1)	75.0 (15.4)	74.6 (16.5)	67.5 (20.7)
** LOCF EP (SD)**	66.9 (20.2)	63.6 (19.2)	67.5 (20.1)	64.0 (19.8)	58.2 (24.0)
** Mean change from BL to LOCF EP (SD)**	−8.8 (19.2)	−10.1 (18.4)	−7.5 (19.4)	−10.6 (21.5)	−9.2 (21.1)
** 95% CI**	−14.1, −3.5	−16.5, −3.7	−13.4, −1.5	−17.4, −3.8	−15.0, −3.5
***p* value^†^**	<0.0001	0.0006	0.0029	0.0007	0.0001
**Mean PANSS Positive Subscale, *n***	53	34	44	41	54
**BL (SD)**	16.2 (5.8)	16.2 (4.5)	15.5 (5.3)	16.5 (5.1)	14.5 (6.4)
** LOCF EP (SD)**	15.1 (7.2)	14.1 (5.9)	14.6 (5.8)	14.2 (5.6)	13.1 (6.5)
** Mean change from BL to LOCF EP (SD)**	−1.1 (5.9)	−2.1 (5.7)	−0.9 (5.1)	−2.3 (5.7)	−1.4 (5.3)
** 95% CI**	−2.7, 0.6	−4.1, −0.2	−2.4, 0.7	−4.1, −0.5	−2.8, 0.1
*** p* value^†^**	0.0344	0.0035	0.1777	0.0146	0.0539
**Mean PANSS Negative Subscale, *n***	53	34	44	41	54
** BL (SD)**	22.3 (4.7)	20.5 (4.4)	23.0 (5.5)	21.2 (6.5)	19.5 (6.8)
** LOCF EP (SD)**	18.8 (5.5)	17.5 (4.8)	19.3 (5.7)	17.6 (6.7)	15.6 (6.6)
** Mean change from BL to LOCF EP (SD)**	−3.5 (6.1)	−3.0 (4.7)	−3.7 (5.6)	−3.6 (6.5)	−3.9 (6.0)
**95% CI**	−5.2, −1.9	−4.6, −1.3	−5.4, −2.0	−5.6, −1.6	−5.6, −2.3
*** p* value^†^**	<0.0001	0.0004	<0.0001	<0.0001	<0.0001
**Mean PANSS General Psychopathology Subscale, *n***	53	34	44	41	54
**BL (SD)**	37.2 (8.4)	37.0 (7.8)	36.5 (8.4)	36.9 (8.8)	33.5 (10.5)
** LOCF EP (SD)**	32.9 (10.9)	32.0 (10.0)	33.6 (11.3)	32.3 (9.8)	29.5 (13.3)
** Mean change from BL to LOCF EP (SD)**	−4.2 (10.3)	−5.0 (9.5)	−2.9 (11.8)	−4.6 (11.5)	−3.9 (12.2)
** 95% CI**	−7.0, −1.4	−8.3, −1.7	−6.5, 0.7	−8.3, −1.0	−7.3, −0.6
*** p* value^†^**	0.0001	0.0007	0.0072	0.0100	0.0017
**Mean PANSS Marder Anxiety/Depression Subscale^‡^, *n***	53	34	44	41	54
** BL (SD)**	9.1 (3.4)	9.5 (3.4)	8.5 (2.7)	9.0 (3.0)	8.9 (3.3)
** LOCF EP (SD)**	7.9 (3.4)	7.5 (3.0)	7.7 (3.3)	7.4 (2.6)	7.2 (3.5)
** Mean change from BL to LOCF EP (SD)**	−1.2 (3.0)	−2.1 (3.2)	−0.8 (3.0)	−1.6 (3.2)	−1.7 (3.4)
** 95% CI**	−2.0, −0.3	−3.2, −0.9	−1.7, 0.1	−2.6, −0.6	−2.6, −0.8
*** p* value^†^**	0.0015	0.0002	0.0840	0.0019	0.0003
**Mean CGI-S score, *n***	53	34	44	41	55
** BL (SD)**	4.2 (0.9)	3.9 (0.8)	4.0 (1.0)	4.1 (1.0)	3.7 (1.2)
** LOCF EP (SD)**	3.8 (1.0)	3.4 (1.0)	3.6 (1.3)	3.6 (1.2)	3.4 (1.3)
** Mean change from BL to LOCF EP (SD)**	−0.4 (1.1)	−0.4 (0.9)	−0.4 (0.9)	−0.5 (1.2)	−0.4 (1.2)
** 95% CI**	−0.7, −0.1	−0.7, −0.1	−0.7, −0.1	−0.9, −0.1	−0.7, −0.0
*** p* value^†^**	0.0076	0.0089	0.0134	0.0065	0.0568

* Only patients with a valid BL measurement and at least one follow-up assessment were included.

† Within-group difference was tested using the Wilcoxon signed-rank test.

‡ Other Marder Factor scores were recorded but are not presented here.

BL: baseline; CGI-S: Clinical Global Impression-Severity; CI: confidence interval; EP, endpoint; LOCF: last observation carried forward; PANSS: Positive and Negative Syndrome Scale; SD: standard deviation.

There was a statistically significant and clinically relevant improvement in the negative and general psychopathology PANSS subscale scores from BL to LOCF EP for all patients, regardless of the LAT from which they were switched (Table 3). There was also a statistically significant improvement in the PANSS positive subscale score for patients switched from Hal-Dec (−1.1; *p* = 0.0344), Fpt-Dec (−2.1; *p* = 0.0035) and Zuc-Dec (−2.3; *p* = 0.0146) and a trend towards improvement for patients switched from RLAT (−1.4.; *p* = 0.0539) (Table 3).

At LOCF EP, 53.7% (Zuc-Dec), 54.7% (Hal-Dec), 59.1% (Flu-Dec), 61.8% (Fpt-Dec) and 61.1% (RLAT) of patients had a ⩾20% improvement in PANSS total score when switched to PP, and 17.0% (Hal-Dec) to 31.5% (RLAT) of patients achieved a ⩾50% improvement in their PANSS total score at LOCF EP.

**Figure 3.**
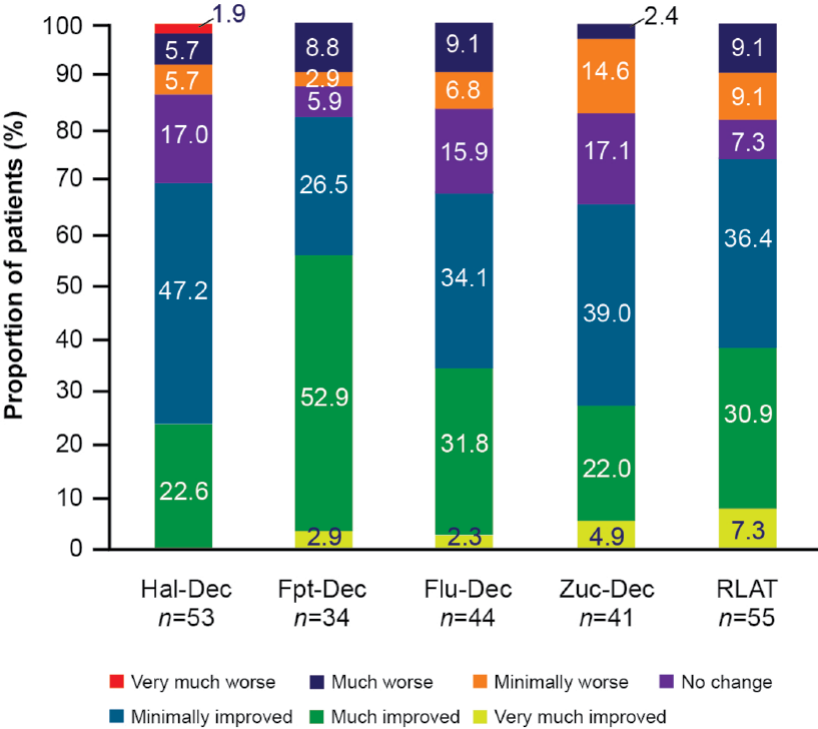
Frequency distribution of CGI-C scores at LOCF endpoint. CGI-C: Clinical Global Impression-Change; Flu-Dec: fluphenazine decanoate; Fpt-Dec: flupentixol decanoate; Hal-Dec: haloperidol decanoate; LOCF: last observation carried forward; RLAT: risperidone long-acting injectable therapy; Zuc-Dec: zuclopenthixol decanoate.

There was a significant reduction in disease severity and improvement in CGI-S total score from BL to LOCF EP in the groups switched from Hal-Dec (*p* = 0.0076), Fpt-Dec (*p* = 0.0089), Flu-Dec (*p* = 0.0134) and Zuc-Dec (*p* = 0.0065) and a trend towards improvement in the group switched from RLAT (*p* = 0.0568) (Table 3). Across all groups, there was an increase from BL to LOCF EP in the proportion of patients who were categorized as mildly ill or less, as assessed by CGI-S score. Furthermore, between 65.9% (Zuc-Dec) and 82.4% (Fpt-Dec) of patients were at least minimally improved, and between 22.6% (Hal-Dec) and 55.9% (Fpt-Dec) were much or very much improved at LOCF EP, as assessed by the frequency distribution of mean CGI-C scores (Figure 3).

There was an improvement in patients’ subjective well-being (SWN-S total score) in all groups, which reached statistical significance in patients switching from Hal-Dec, Fpt-Dec and Zuc-Dec (*p* < 0.05), while patients switching from Fpt-Dec showed a statistically significant improvement in three of the five subscales (*p* < 0.05) (Table 4; Appendix II, supplementary data). A significant improvement in the four TSQM domain scores (effectiveness, side effects, convenience, and global satisfaction) was observed in patients switched from Hal-Dec and Fpt-Dec (*p* = 0.0316 to *p* < 0.0001), indicating improved patient satisfaction compared with BL medication (Appendix III, supplementary data). Convenience and side effects domain scores significantly increased in patients switching from Flu-Dec and Zuc-Dec (*p* = 0.0406 to *p* = 0.0011), while a significant improvement in side effects score was observed in patients switching from RLAT (*p* = 0.0371). There was a significant increase in score for quality of sleep and decrease in score for drowsiness in patients switching from Fpt-Dec (*p* = 0.0004, *p* = 0.0019) and RLAT (*p* = 0.0166, *p* = 0.0292, respectively) (Table 4).

**Table 4.** Subjective well-being, treatment satisfaction, sleep quality and daytime drowsiness.*

	Patients switched from:				
	Conventional depot				RLAT
	Hal-Dec	Fpt-Dec	Flu-Dec	Zuc-Dec	
**Mean SWN-S total score, *n***	46	33	43	37	47
** BL (SD)**	83.7 (12.5)	83.5 (18.6)	81.0 (17.2)	83.0 (15.3)	80.8 (22.2)
** LOCF EP (SD)**	86.9 (15.6)	91.7 (18.5)	83.9 (18.3)	87.4 (17.2)	84.4 (20.0)
** Mean change from BL to LOCF EP (SD)**	3.2 (13.6)	8.3 (17.5)	2.9 (15.5)	4.3 (14.8)	3.6 (15.7)
** 95% CI**	−0.8, 7.2	2.1, 14.5	−1.9, 7.7	−0.6, 9.3	−1.0, 8.2
*** p* value^†^**	0.0356	0.0175	0.3292	0.0335	0.0814
**Mean TSQM total global satisfaction score, *n***	43	31	40	31	44
** BL (SD)**	53.3 (22.8)	54.1 (19.4)	52.5 (20.5)	54.4 (15.6)	61.7 (28.0)
** LOCF EP (SD)**	69.4 (18.1)	72.8 (22.2)	56.6 (28.8)	61.9 (30.1)^¶^	62.5 (29.1)
**Mean change from BL to LOCF EP (SD)**	16.1 (24.3)	18.7 (26.5)	4.1 (35.0)	7.9 (32.0)	0.8 (28.5)
** 95% CI**	8.6, 23.6	8.9, 28.4	−7.1, 15.3	−4.1, 19.8	−7.9, 9.5
*** p* value^†^**	<0.0001	0.0003	0.5181	0.1444	0.5857
**Quality of sleep score^‡^, *n***	52	34	44	41	52
** BL (SD)**	6.9 (2.3)	5.9 (3.0)	6.2 (3.0)	7.2 (2.3)	6.6 (2.7)
** LOCF EP (SD)**	7.4 (2.5)	7.5 (2.4)	7.3 (2.4)	7.3 (2.1)	7.5 (2.2)
** Mean change from BL to LOCF EP (SD)**	0.6 (2.7)	1.6 (3.4)	1.1 (3.0)	0.1 (2.7)	0.9 (2.5)
** 95% CI**	−0.2, 1.3	0.5, 2.8	0.2, 2.0	−0.8, 1.0	0.2, 1.6
*** p* value^†^**	0.0912	0.0004	0.0365	0.6796	0.0166
**Daytime drowsiness score^§^, *n***	52	34	44	40	52
** BL (SD)**	3.5 (2.8)	4.6 (3.4)	4.2 (2.6)	3.3 (2.4)	4.3 (3.4)
** LOCF EP (SD)**	2.7 (2.7)	3.0 (2.7)	3.3 (2.5)	3.2 (2.6)	3.3 (3.1)
**Mean change from BL to LOCF EP (SD)**	−0.8 (3.3)	−1.6 (2.7)	−0.9 (3.3)	−0.1 (2.4)	−1.0 (3.4)
** 95% CI**	−1.7, 0.1	−2.6, −0.7	−1.9, 0.1	−0.9, 0.7	−1.9, −0.0
*** p* value^†^**	0.0649	0.0019	0.0964	0.7356	0.0292

* Only patients with a valid BL measurement and at least one valid follow-up assessment were included.

† Within-group difference was tested using the Wilcoxon signed-rank test.

‡ A higher score indicates improvements in the quality of sleep.

§ A lower score indicates improvements in the level of drowsiness.

¶ *n* = 30.

BL: baseline; CI: confidence interval; EP, endpoint; LOCF: last observation carried forward; SD: standard deviation; SWN: Subjective Well-being under Neuroleptics; TSQM: Treatment Satisfaction Questionnaire for Medication.

### Functioning outcomes

**Figure 4.**
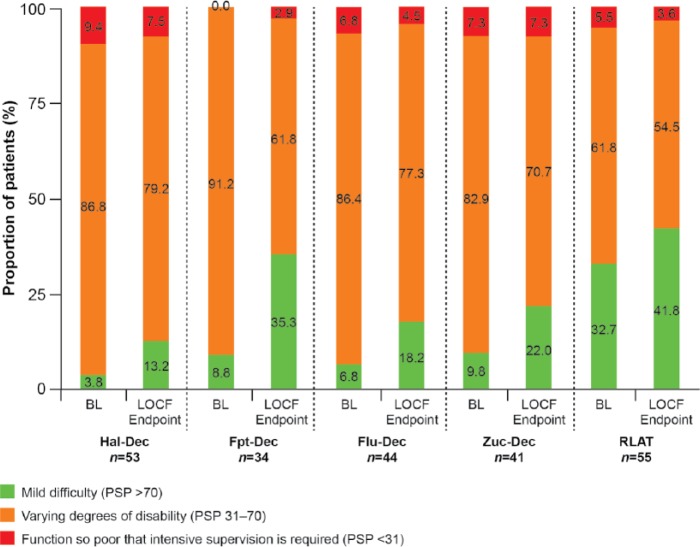
Frequency distribution of PSP total score over time. BL: baseline; Flu-Dec: fluphenazine decanoate; Fpt-Dec: flupentixol decanoate; Hal-Dec: haloperidol decanoate; LOCF: last observation carried forward; PSP: Personal and Social Performance; RLAT: risperidone long-acting injectable therapy; Zuc-Dec: zuclopenthixol decanoate.

At LOCF EP, there was a statistically significant increase from BL to EP in mean PSP total scores (SD) in all groups switched to PP, ranging from 5.2 (13.0) (*p* = 0.0013) and 5.2 (15.3) (*p* = 0.0163) for patients switched from Hal-Dec and RLAT, respectively, to 6.4 (15.2) (*p* = 0.0013) for patients switched from Zuc-Dec (Table 5). The PSP domain scores of socially useful activities including work and study (*p* = 0.0217 to *p* < 0.0001) and personal and social relationships were significantly improved in all groups (*p* = 0.0421 to *p* < 0.0001) (Appendix IV, supplementary data). The self-care domain was significantly improved (mean change: −0.3 to −0.5; *p* value: 0.0029 to 0.0279) in all but the group of patients switched from RLAT where there was a trend towards improvement (mean change: −0.2; *p* = 0.0904). The proportion of patients with a PSP score >70 (indicating mild to no functional impairment) increased in all groups (Figure 4).

**Table 5.** Functioning outcomes.*

	Patients switched from:				
	Conventional depot				RLAT
	Hal-Dec	Fpt-Dec	Flu-Dec	Zuc-Dec	
**Mean PSP score, *n***	53	34	44	41	55
** BL (SD)**	48.7 (12.5)	59.6 (11.2)	53.5 (12.2)	52.9 (15.6)	60.1 (17.9)
** LOCF EP (SD)**	53.8 (14.9)	65.7 (14.3)	59.5 (14.3)	59.2 (15.8)	65.3 (18.1)
**Mean change from BL to LOCF EP (SD)**	5.2 (13.0)	6.1 (14.9)	6.0 (11.6)	6.4 (15.2)	5.2 (15.3)
** 95% CI**	1.6, 8.8	0.9, 11.3	2.5, 9.5	1.6, 11.2	1.0, 9.3
*** p* value^†^**	0.0013	0.0071	<0.0001	0.0013	0.0163
**Mean Mini-ICF-APP total score, *n***	47	33	43	38	52
** BL (SD)**	23.0 (7.7)	20.5 (8.3)	21.7 (8.3)	21.1 (9.0)	18.4 (10.1)
** LOCF EP (SD)**	19.6 (9.2)	15.8 (8.3)	20.0 (8.7)	18.6 (8.8)	15.7 (10.4)
**Mean change from BL to LOCF EP (SD)**	−3.3 (7.6)	−4.6 (7.0)	−1.7 (6.3)	−2.5 (9.0)	−2.6 (6.7)
** 95% CI**	−5,6, −1.1	−7.1, −2.1	−3.7, 0.2	−5.4, 0.5	−4.5, −0.8
*** p* value^†^**	0.0012	0.0001	0.0096	0.1231	0.0137

* Only patients with a valid BL measurement and at least one valid follow-up assessment were included.

† Within-group difference was tested using the Wilcoxon signed-rank test.

BL: baseline; CI: confidence interval; EP, endpoint; LOCF: last observation carried forward; Mini-ICF-APP: mini International Classification of Functionality, Disability and Health (ICF) Rating for Activity and Participation Disorders in Psychological Illnesses; PSP: Personal and Social Performance; SD: standard deviation.

There was a decrease (i.e. improvement) from BL in the Mini-ICF-APP total score at LOCF EP, which varied from −1.7 (Flu-Dec) to −4.6 (Fpt-Dec) and was statistically significant in all patient groups except those switched from Zuc-Dec (Table 5).

### Tolerability and safety

**Figure 5.**
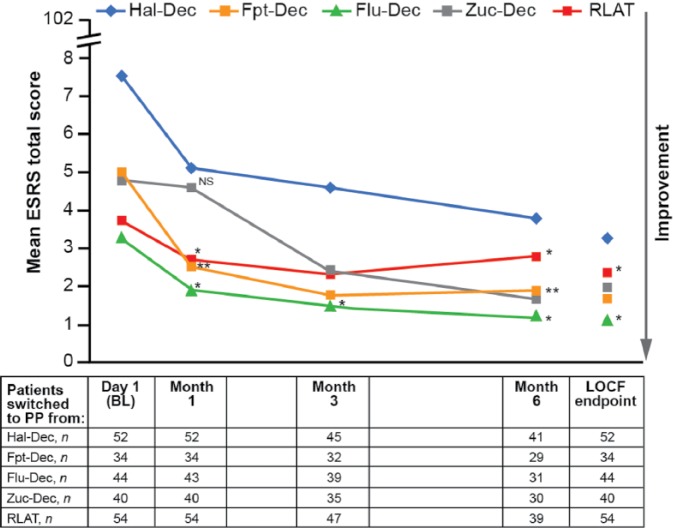
ESRS total score over time (safety ITT population). *p* values < 0.0001 vs. BL for all data points except for NS, non-significant; *p < 0.05; **p < 0.001 Within-group difference was tested using the Wilcoxon signed-rank test. BL: baseline; ESRS: Extrapyramidal Symptom Rating Scale; Flu-Dec: fluphenazine decanoate; Fpt-Dec: flupentixol decanoate: Hal-Dec, haloperidol decanoate; ITT: intent-to-treat; LOCF: last observation carried forward; PP: paliperidone palmitate; RLAT: risperidone long-acting injectable therapy; Zuc-Dec: zuclopenthixol decanoate.

The mean ESRS scores (SD) at BL ranged from 3.3 (5.4) for patients switched from Flu-Dec to 7.5 (8.1) for patients switched from Hal-Dec (Figure 5). Statistically significant reductions in ESRS total score were observed in all groups at LOCF EP, ranging from −1.2 for RLAT to −4.1 for Hal-Dec (*p* values ranging from <0.0001 to 0.0045), indicating improvement in EPMS (Figure 5). In patients switched from Hal-Dec, Flu-Dec and Zuc-Dec, there was a clinically meaningful reduction in the use of concomitant anticholinergic medication from BL to LOCF EP (24.5 to 9.4%; 11.4 to 2.3% and 31.0 to 19.0%, respectively) (Table 6), suggesting further improvement of EPMS.

**Table 6.** Use of concomitant medication.

	Patients switched from:				
	Conventional depot				RLAT(*n* = 56)
	Hal-Dec (*n* = 53)	Fpt-Dec (*n* = 35)	Flu-Dec (*n* = 44)	Zuc-Dec (*n* = 42)	
**Number (%) of patients using benzodiazepines**					
**At BL**	18 (34.0)	3 (8.6)	9 (20.5)	14 (33.3)	12 (21.4)
**Newly initiated during study**	14 (26.4)	3 (8.6)	10 (22.7)	10 (23.8)	9 (16.1)
**At LOCF EP**	15 (28.3)	3 (8.6)	8 (18.2)	14 (33.3)	11 (19.6)
**At 6 months for completers**	12 (29.3)	1 (3.3)	5 (16.1)	10 (33.3)	8 (20.0)
**Number (%) of patients using anticholinergics**					
**At BL**	13 (24.5)	2 (5.7)	5 (11.4)	13 (31.0)	4 (7.1)
**Newly initiated during study**	7 (13.2)	2 (5.7)	1 (2.3)	5 (11.9)	3 (5.4)
**At LOCF EP**	5 (9.4)	3 (8.6)	1 (2.3)	8 (19.0)	3 (5.4)
**At 6 months for completers**	5 (12.2)	1 (3.3)	1 (3.2)	5 (16.7)	2 (5.0)

BL: baseline; EP, endpoint; LOCF: last observation carried forward.

TEAEs affecting ⩾5% of patients in any group are summarized in Table 7. Overall, 50.9%, 40.0%, 40.9%, 54.8% and 62.5% of patients switching from Hal-Dec, Fpt-Dec, Flu-Dec, Zuc-Dec and RLAT, respectively, experienced at least one TEAE. In all groups the majority of TEAEs (83.3–97.9%) were rated as mild or moderate in intensity. Most TEAEs across groups resulted in no change in dose (56.7% [Fpt-Dec] to 79.2% [RLAT]), required no concomitant treatment (26.7% [Fpt-Dec] to 54.9% [Zuc-Dec]), and were considered resolved (63.3% [Fpt-Dec] to 84.4% [RLAT]) by the end of the study (Table 7). No deaths occurred in patients switched from previous unsuccessful treatment with another LAT to PP.

**Table 7.** Tolerability and safety (ITT safety population; *n* = 230).

	Patients switched from:				
	Conventional depot				RLAT (*n* = 56)
	Hal-Dec (*n* = 53)	Fpt-Dec (*n* = 35)	Flu-Dec (*n* = 44)	Zuc-Dec (*n* = 42)	
**TEAEs occurring in ⩾5% of patients in any switching group**					
**Number of patients with ⩾1 TEAE**	27 (50.9)	14 (40.0)	18 (40.9)	23 (54.8)	35 (62.5)
**Injection site pain**	2 (3.8)	1 (2.9)	4 (9.1)	3 (7.1)	4 (7.1)
**Weight increased**	0 (0)	2 (5.7)	2 (4.5)	0 (0)	1 (1.8)
**Constipation**	0 (0)	0 (0)	0 (0)	0 (0)	3 (5.4)
**Headache**	3 (5.7)	0 (0)	2 (4.5)	5 (11.9)	4 (7.1)
**Somnolence**	1 (1.9)	0 (0)	1 (2.3)	4 (9.5)	3 (5.4)
**Hallucination**	0 (0)	2 (5.7)	0 (0)	1 (2.4)	0 (0)
**Insomnia**	2 (3.8)	4 (11.4)	4 (9.1)	4 (9.5)	1 (1.8)
**Psychotic disorders**	3 (5.7)	3 (8.6)	3 (6.8)	4 (9.5)	6 (10.7)
**Anxiety**	6 (11.3)	0 (0)	0 (0)	2 (4.8)	4 (7.1)
**Schizophrenia**	3 (5.7)	0 (0)	3 (6.8)	1 (2.4)	4 (7.1)
**Suicidal ideation**	1 (1.9)	2 (5.7)	0 (0)	1 (2.4)	0 (0)
**Outcomes following TEAEs, *n* (%)**					
**Dose not changed**	49 (68.1)	17 (56.7)	28 (70.0)	52 (73.2)	76 (79.2)
**Concomitant medication started after TEAE**	36 (50.0)	8 (26.7)	20 (50.0)	39 (54.9)	33 (34.4)
**Considered resolved**	57 (79.2)	19 (63.3)	26 (65.0)	54 (76.1)	81 (84.4)

TEAE: treatment-emergent adverse event.

Potentially prolactin-related TEAEs were uncommon. Across all previous treatment groups (RLAT and conventional depots combined), 3.0% of patients reported at least one potentially prolactin-related TEAE. MedDRA-preferred terms that were considered potentially prolactin-related AEs were amenorrhea (RLAT: 1.8%), erectile dysfunction (Flu-Dec: 2.3%), galactorrhea (Fpt-Dec: 2.9%; Flu-Dec: 2.3%), loss of libido (Zuc-Dec: 2.4%) and sexual dysfunction (Hal-Dec: 1.9%; RLAT: 1.8%). 5.7% and 2.3% of patients switching from Hal-Dec and Flu-Dec, respectively, reported hyperprolactinaemia.

## Discussion

This is the first study designed to explore treatment response, safety and tolerability of PP in patients directly switching from previous treatment with either RLAT or conventional depot APs. Data support results provided by previous fixed-dose, randomized controlled clinical trials in which the efficacy of PP in the treatment of schizophrenia has been demonstrated. In this study, the primary efficacy analysis (change in PANSS total score) showed that statistically significant and clinically meaningful improvements in the symptoms of schizophrenia were achieved in non-acute patients switched to PP following previous unsuccessful treatment with LATs. Results of the primary analysis were further supported by improvements in disease severity, psychosocial functioning, relevant ability domains of activation and participation, and treatment satisfaction.

The PALMFlexS study has several strengths, namely closely mimicking daily clinical practice compared with RCTs, enrolling patients with relevant comorbidities, comedications and substance abuse and allowing flexible dosing enabling optimization of treatment based on patients’ individual needs. This is reflected by the range of maintenance doses used and in the variation of the dose changes required across the groups during the study, indicating that treating physicians adjusted the dose depending on the individual patient’s needs to ensure optimal symptom control and achieve better patient outcomes.

Furthermore, it is possible that patients in an open-label flexible dose study may continue treatment for longer than achievable otherwise (as evidenced by >70% of patients completing this 6-month study), providing more meaningful data over the duration of the study.

Continuous treatment is particularly important for patients with schizophrenia, because disruption of long-term treatment increases the risk of relapse and hospitalization (Kane, 2013). A number of studies have suggested that treatment interruption is directly linked to relapse even after long-term successful treatment, in turn contributing to persistence of symptoms and loss of gains in functioning and quality of life (Leucht et al., 2012; Masand et al., 2009; Morken et al., 2008; Velligan et al., 2009; Wiersma et al., 1998). Switching to LAT may improve adherence to treatment in patients with schizophrenia (Cañas et al., 2013) due to the transparency of delivery of medication (Kane, 2007). Most schizophrenia treatment guidelines (Buchanan et al., 2010; National Institute for Health and Care Excellence, 2014) recommend the use of LAT over oral AP agents in patients who are covertly non-adherent to treatment or in those who would prefer such treatment (Kane and Garcia-Ribera, 2009). No preference is given as to the choice of agent for the maintenance treatment of schizophrenia, but it is recommended that clinical response and adverse effects, both those potentially related to the new drug and those experienced with current or previous medications, should be taken into account (Kane and Garcia-Ribera, 2009; National Institute for Health and Care Excellence, 2014). It should be considered that the observed efficacy benefits of treatment with PP may be due to continuous treatment through improved adherence to AP medication as a result of the increased interaction with healthcare professionals during the course of the study. Patients who switched from RLAT (mean biweekly dose of 50.2 mg) received a mean modal monthly maintenance dose of PP of 104.2 mg eq., which is broadly in line with recommendations to attain similar paliperidone exposure at steady state on switching treatment (50 mg biweekly RLAT is the equivalent of approximately 100 mg eq. once monthly PP) (Xeplion SmPC, 2015). Therefore, in these patients, the improvements in efficacy are unlikely to be due to a change in dose (detailed information on dosing is provided in Table 2). Patient preference must also play a role in treatment selection.

Several factors might motivate a patient or physician to switch from a conventional depot AP to an atypical LAT, including improved effect on psychopathology (negative and depressive symptoms) (Leucht et al., 2009), tolerability issues, such as reduced injection site reactions and pain (in switching from an oil-based to an aqueous formulation), lower risk of EPMS and tardive dyskinesia (Gopal et al., 2011), sedation and prolactin levels and reduced impact on sexual function (Montalvo et al., 2013), as well as the practical advantage of the monthly administration schedule (Carter, 2012). The benefits of such a switch have been substantiated by the findings of this study, in which patients with schizophrenia deemed stable by their treating physician and where non-adherence to prior AP treatment has not been a factor, when switched to flexibly dosed PP following previous unsuccessful treatment with either RLAT or a conventional depot AP, showed significant and clinically relevant improvements in clinical symptoms. This finding is of particular clinical relevance given that more than half of the patients showed further clinical improvement in positive, negative and depressive symptoms, demonstrating that patients who are considered clinically stable on treatment still can experience symptom improvement upon switching. Treatment response, as indicated by the significant improvement in psychotic symptoms, was also associated with clinically meaningful improvements in patient functioning. In particular, patients in this study showed some significant improvements in maintaining or building new relationships and performing meaningful activities, specific domains in which patients with schizophrenia often experience major deficits.

In this patient population, PP was generally safe and well tolerated, with no new safety signals compared with previous RCTs. Switching from a conventional depot AP to PP in this trial was associated with a clinically meaningful improvement of EPMS. This is of relevance because patients are often reluctant to take LATs due to the risk of EPMS. In addition, reducing EPMS also decreases the risk of developing tardive dyskinesia. In contrast to patients switched from Hal-Dec and Fpt-Dec, who showed significant improvement across all four domains of treatment satisfaction, patients switched from Flu-Dec and Zuc-Dec showed significant improvement in side effects and convenience, whereas those patients switching from RLAT the observed improvement was statistically significant only in side effect score. When switching from RLAT to PP, the results may in part reflect the distinct pharmacokinetic profiles of PP and RLAT, whereby there is a transient decrease in plasma levels approximately 8–10 weeks following the last RLAT injection in those patients switched to PP (Samtani et al., 2011). In individual patients, this transient decrease may be associated with a transient worsening of symptoms. In such a case of clinical deterioration, temporary treatment, for example with an oral antipsychotic, may be clinically indicated.

Overall, data from the current study support results provided by previous fixed-dose, RCTs in which the efficacy, tolerability and safety of PP in the treatment of schizophrenia has been demonstrated. These data also support those observed in non-acute patients and acute patients switched from oral APs in the PALMFlexS trial (Hargarter et al., 2015; Schreiner et al., 2014).

Results from this study should also be considered in light of some methodological limitations. As a non-comparative study, no conclusions can be reached regarding the relative impact of previous treatment (RLAT/conventional depot) from which patients switched to PP compared with other APs. Also, there were some numerical between-group differences in age, weight, BMI, gender and psychotic symptoms which may limit the comparability of data between groups. One related question may be whether the difference observed in baseline PANSS total score between RLAT and conventional depots was associated with better symptom control in patients receiving RLAT (as suggested, for example, by Rubio et al., 2006) or whether the difference was due to a selection bias. In addition, the open-label nature of the study may have subjected the results to bias. However, pragmatic open-label studies are considered valuable because they add clinically relevant information on treatment effectiveness, complementing the evidence provided by RCTs (Kirson et al., 2013). The current study was designed to mimic situations closely, including dosing and switching, as they occur in naturalistic clinical settings, and to provide clinical experience of long-term treatment with PP, drawing on the clinical judgment of physicians to evaluate tolerability, safety and treatment response in order to access the most appropriate dose of PP.

In conclusion, these data illustrate that non-acute patients with schizophrenia considered stable by their treating physician show clinically relevant improvement in clinical symptoms, functioning and relevant side effects when switched from previous RLAT or frequently used conventional depot APs to PP.

## Supplementary Material

Supplementary material
