# Supplementary material for: Paliperidone palmitate in non-acute patients with schizophrenia previously unsuccessfully treated with risperidone long-acting therapy or frequently used conventional depot antipsychotics
Source: J Psychopharmacol. 2015 Aug;29(8):910–22. doi: 10.1177/0269881115586284 (PMC4512527; doi:10.1177/0269881115586284)
Supplement: Supplementary material [file JOP586284_Appendix.pdf]

Appendix I. Participants

Trial participants were recruited from 160 centres across Austria, Belgium, Croatia, Denmark, Estonia, France, Germany, Greece, Hungary, Israel, Italy, Latvia, Lithuania, the Netherlands, Portugal, Spain, Sweden, Switzerland, Turkey, Ukraine and the United Kingdom.

Appendix II. SWN subscale scores.*

	Hal-Dec (*n* = 53)		Fpt-Dec (*n* = 34)		Flu-Dec (*n* = 44)		Zuc-Dec (*n* = 41)		RLAT (*n* = 55)	
	**Actual value**	**Change from BL**	**Actual value**	**Change from BL**	**Actual value**	**Change from BL**	**Actual value**	**Change from BL**	**Actual value**	**Change from BL**
**Mental functioning**										
**BL visit Day 1**										
n	46		33		43		37		47	
**Mean**	15.7		15.6		15.3		15.8		15.7	
**SD**	3.80		5.20		4.60		3.81		5.04	
**95% CI**	14.5, 16.8		13.8, 17.5		13.9, 16.7		14.5, 17.0		14.2, 17.2	
**Month 6 LOCF EP visit**										
**Mean**	16.2	0.6	17.9	2.2	16.0	0.7	16.4	0.7	16.4	0.7
**SD**	4.42	4.79	3.97	4.57	4.28	3.68	4.00	3.75	4.57	4.37
**95% CI**	14.9, 17.5	-0.9, 2.0	16.5, 19.3	0.6, 3.9	14.7, 17.4	-0.4, 1.9	15.1, 17.8	-0.6, 1.9	15.0, 17.7	-0.6, 1.9
***p* value**†		0.4046		0.0076		0.2525		0.1447		0.2401
**Self-control**										

**BL visit Day 1**										
n	46		33		43		37		47	
**Mean**	17.8		17.5		16.7		16.8		16.6	
**SD**	3.03		3.46		3.26		3.65		4.67	
**95% CI**	16.9, 18.7		16.2, 18.7		15.7, 17.7		15.5, 18.0		15.3, 18.0	
**Month 6 LOCF EP visit**										
**Mean**	18.0	0.2	18.6	1.2	18.0	1.2	18.4	1.6	17.1	0.5
**SD**	3.65	3.63	3.83	4.81	3.38	3.13	3.34	3.72	4.42	4.30
**95% CI**	16.9, 19.1	-0.9, 1.3	17.3, 20.0	-0.5, 2.9	16.9, 19.0	0.3, 2.2	17.2, 19.5	0.4, 2.8	15.8, 18.4	-0.8, 1.7
***p* value†**		0.6036		0.2655		0.0235		0.0097		0.2082
**Emotional regulation**										
**BL visit Day 1**										
n	46		33		43		37		47	
**Mean**	16.7		16.8		16.1		16.8		16.8	
**SD**	3.29		3.73		3.94		3.40		4.65	
**95% CI**	15.7,		15.5,		14.9,		15.7,		15.5,	

	17.6		18.2		17.3		17.9		18.2	
**Month 6 LOCF EP visit**										
**Mean**	17.1	0.4	18.5	1.7	16.7	0.6	17.9	1.2	17.0	0.2
**SD**	4.06	4.29	4.35	3.57	4.26	3.99	3.94	3.88	4.55	3.33
**95% CI**	15.9, 18.3	-0.9, 1.7	17.0, 20.1	0.4, 3.0	15.4, 18.0	-0.6, 1.8	16.6, 19.3	-0.1, 2.5	15.7, 18.3	-0.8, 1.1
***p* value**[†]		0.1994		0.0122		0.3395		0.0457		0.7878
**Physical functioning**										
**BL visit Day 1**										
n	46		33		43		37		47	
**Mean**	17.5		16.8		16.6		17.2		15.9	
**SD**	3.22		4.85		4.63		4.12		5.27	
**95% CI**	16.6, 18.5		15.1, 18.6		15.2, 18.1		15.8, 18.6		14.4, 17.5	
**Month 6 LOCF EP visit**										
**Mean**	18.7	1.2	18.4	1.6	16.8	0.1	17.5	0.4	17.3	1.3
**SD**	3.50	3.39	4.66	4.62	5.17	4.66	4.77	4.00	4.55	4.07
**95% CI**	17.6,	0.1, 2.2	16.8,	-0.1, 3.2	15.2,	-1.3, 1.6	16.0,	-1.0, 1.7	15.9,	0.1, 2.5

	19.7		20.1		18.4		19.1		18.6	
***p* value†**		0.0111		0.0697		0.6274		0.5838		0.0209
**Social integration**										
**BL visit Day 1**										
n	46		33		43		37		47	
**Mean**	16.1		16.7		16.3		16.5		15.7	
**SD**	3.62		4.18		3.62		3.60		5.00	
**95% CI†**	15.1, 17.2		15.2, 18.1		15.2, 17.4		15.3, 17.7		14.2, 17.1	
**Month 6 LOCF EP visit**										
**Mean**	17.0	0.8	18.2	1.6	16.4	0.2	17.1	0.5	16.6	1.0
**SD**	3.56	3.83	4.03	3.63	3.89	3.95	4.41	3.72	4.04	4.01
**95% CI**	15.9, 18.0	-0.3, 2.0	16.8, 19.7	0.3, 2.9	15.2, 17.6	-1.1, 1.4	15.6, 18.6	-0.7, 1.8	15.4, 17.8	-0.2, 2.1
***p* value†**		0.2469		0.0169		0.8470		0.4007		0.1655

*Only patients with a valid BL measurement and at least one valid follow-up assessment were included

†Within-group difference was tested using the Wilcoxon signed-rank test

BL: baseline; CI: confidence interval; EP, Endpoint; LOCF: last observation carried forward; SD: standard deviation; SWN: Subjective Well-being under Neuroleptics

Appendix III. TSQM subscale scores.*

	Hal-Dec (*n* = 53)		Fpt-Dec (*n* = 34)		Flu-Dec (*n* = 44)		Zuc-Dec (*n* = 41)		RLAT (*n* = 55)	
	**Actual value**	**Change from BL**	**Actual value**	**Change from BL**	**Actual value**	**Change from BL**	**Actual value**	**Change from BL**	**Actual value**	**Change from BL**
**Effectiveness**										
BL visit Day 1										
n	43		32		40		31		44	
**Mean**	54.5		59.7		52.5		57.2		57.1	
**SD**	20.65		19.19		18.23		13.24		29.87	
**95% CI**	48.2, 60.9		52.8, 66.6		46.7, 58.3		52.3, 62.0		48.1, 66.2	
**Month 6 LOCF EP visit**										
**Mean**	66.1	11.6	71.0	11.3	59.0	6.5	64.5	7.3	61.7	4.6
**SD**	21.44	22.61	19.39	28.49	24.19	30.99	22.16	24.82	29.33	26.21
**95% CI**	59.6, 72.7	4.7, 18.6	64.0, 78.0	1.0, 21.6	51.3, 66.8	-3.4, 16.4	56.4, 72.6	-1.8, 16.5	52.8, 70.7	-3.4, 12.6
***p* value**†		0.0009		0.0165		0.1824		0.1343		0.3449
**Side effects**										

**BL visit Day 1**										
n	43		31		40		31		44	
**Mean**	70.8		76.4		67.7		73.0		71.4	
**SD**	30.27		34.22		29.28		28.29		30.98	
**95% CI**	61.5, 80.1		63.9, 89.0		58.3, 77.0		62.6, 83.4		62.0, 80.9	
**Month 6 LOCF EP visit**										
n	43	43	31	31	40	40	31	31	44	44
**Mean**	93.8	23.0	96.0	19.6	87.2	19.5	88.9	15.9	81.8	10.4
**SD**	14.04	32.53	14.93	34.79	23.17	35.10	22.58	36.83	26.20	31.78
**95% CI**	89.4, 98.1	13.0, 33.0	90.5, 101.4	6.8, 32.3	79.8, 94.6	8.3, 30.8	80.6, 97.2	2.4, 29.4	73.9, 89.8	0.7, 20.0
***p* value[†]**		<0.0001		0.0029		0.0011		0.0148		0.0371
**Convenience**										
**BL visit Day 1**										
n	43		31		40		31		44	
**Mean**	63.6		68.5		64.4		61.6		68.2	
**SD**	17.99		16.13		17.92		16.81		23.82	

**95% CI**	58.0, 69.1		62.5, 74.4		58.7, 70.2		55.5, 67.8		60.9, 75.4	
**Month 6 LOCF EP visit**										
n	43	43	31	31	40	40	31	31	44	44
**Mean**	72.5	8.9	76.9	8.4	72.5	8.1	71.0	9.3	72.5	4.3
**SD**	14.19	17.99	19.14	19.87	17.70	24.97	17.26	21.91	22.64	21.06
**95% CI**	68.1, 76.8	3.4, 14.5	69.9, 83.9	1.1, 15.7	66.8, 78.2	0.1, 16.0	64.6, 77.3	1.3, 17.4	65.6, 79.4	-2.1, 10.7
***p* value**[†]		0.0023		0.0316		0.0406		0.0237		0.1456
**Global satisfaction**										
**BL visit Day 1**										
n	43		31		40		31		44	
**Mean**	53.3		54.1		52.5		54.4		61.7	
**SD**	22.77		19.41		20.50		15.61		27.95	
**95% CI**	46.3, 60.3		47.0, 61.3		45.9, 59.1		48.7, 60.1		53.2, 70.2	
**Month 6 LOCF EP visit**										
n	43	43	31	31	40	40	30	30	44	44
**Mean**	69.4	16.1	72.8	18.7	56.6	4.1	61.9	7.9	62.5	0.8

**SD**	18.13	24.30	22.24	26.51	28.82	34.95	30.09	31.96	29.07	28.54
**95% CI**	63.9, 75.0	8.6, 23.6	64.7, 81.0	8.9, 28.4	47.4, 65.8	-7.1, 15.3	50.7, 73.1	-4.1, 19.8	53.7, 71.3	-7.9, 9.5
***p* value**†		<0.0001		0.0003		0.5181		0.1444		0.5857

*Only patients with a valid BL measurement and at least one valid follow-up assessment were included

†Within-group difference was tested using the Wilcoxon signed-rank test

BL: baseline; CI: confidence interval; EP, endpoint; LOCF: last observation carried forward; SD: standard deviation; TSQM: Treatment Satisfaction Questionnaire for Medication

Appendix IV. PSP subscales.

	Hal-Dec (*n* = 53)		Fpt-Dec (*n* = 34)		Flu-Dec (*n* = 44)		Zuc-Dec (*n* = 41)		RLAT (*n* = 55)	
	**Actual value**	**Change from BL**	**Actual value**	**Change from BL**	**Actual value**	**Change from BL**	**Actual value**	**Change from BL**	**Actual value**	**Change from BL**
**Socially useful activities; including work and study**										
**BL visit Day 1**										
n	53		34		44		41		55	
**Mean**	3.9		3.4		3.5		3.7		3.3	
**SD**	1.15		1.02		0.85		1.17		1.29	
**95% CI**	3.6, 4.2		3.1, 3.8		3.3, 3.8		3.3, 4.1		3.0, 3.7	
**Month 6 LOCF EP visit**										
n	53	53	34	34	44	44	41	41	55	55
**Mean**	3.6	-0.3	2.7	-0.7	3.2	-0.3	3.3	-0.4	2.8	-0.5
**SD**	1.18	0.87	0.87	0.97	1.02	0.81	1.17	1.05	1.26	1.09
**95% CI**	3.3, 3.9	-0.5, -0.1	2.4, 3.0	-1.0, -0.4	2.9, 3.5	-0.6, -0.1	2.9, 3.7	-0.7, -0.1	2.5, 3.2	-0.8, -0.2
***p* value**		0.0174		<0.0001		0.0029		0.0217		0.0012
**Personal and social relationships**										

**BL visit Day 1**										
n	53		34		44		41		55	
**Mean**	3.8		3.1		3.4		3.2		3.0	
**SD**	0.80		1.01		0.79		1.05		1.16	
**95% CI**	3.6, 4.1		2.7, 3.4		3.2, 3.7		2.8, 3.5		2.7, 3.3	
**Month 6 LOCF EP visit**										
n	53	53	34	34	44	44	41	41	55	55
**Mean**	3.3	-0.5	2.6	-0.4	2.9	-0.5	2.8	-0.4	2.7	-0.3
**SD**	1.04	0.97	0.89	1.13	1.00	0.98	1.03	0.97	1.13	1.09
**95% CI**	3.0, 3.6	-0.8, -0.3	2.3, 2.9	-0.8, -0.0	2.6, 3.2	-0.8, -0.2	2.5, 3.1	-0.7, -0.1	2.4, 3.0	-0.6, -0.0
***p* value**		<0.0001		0.0421		0.0003		0.0259		0.0309
**Self-care**										
**BL visit Day 1**										
n	53		34		44		41		55	
**Mean**	2.3		2.0		2.2		2.5		2.3	

**SD**	1.09		0.97		0.90		1.19		1.15	
**95% CI**	2.0, 2.6		1.7, 2.4		1.9, 2.5		2.2, 2.9		2.0, 2.6	
**Month 6 LOCF EP visit**										
n	53	53	34	34	44	44	41	41	55	55
**Mean**	2.0	-0.3	1.6	-0.4	1.9	-0.3	2.0	-0.5	2.1	-0.2
**SD**	1.02	1.22	0.69	0.65	0.70	0.65	1.11	0.95	1.16	1.13
**95% CI**	1.7, 2.3	-0.7, -0.0	1.4, 1.9	-0.6, -0.2	1.7, 2.1	-0.4, -0.1	1.7, 2.4	-0.8, -0.2	1.7, 2.4	-0.5, 0.1
***p* value**		0.0279		0.0029		0.0232		0.0011		0.0904
**Disturbing and aggressive behaviours**										
**BL visit Day 1**										
n	53		34		44		41		55	
**Mean**	1.5		1.5		1.4		1.7		1.5	
**SD**	0.91		0.71		0.70		0.88		0.84	
**95% CI**	1.3, 1.8		1.3, 1.8		1.2, 1.6		1.4, 2.0		1.2, 1.7	
**Month 6 LOCF EP Visit**										

n	53	53	34	34	44	44	41	41	55	55
**Mean**	1.5	-0.1	1.3	-0.2	1.4	-0.0	1.4	-0.2	1.5	0.0
**SD**	0.75	1.03	0.68	0.78	0.73	0.63	0.84	0.99	0.86	0.94
**95% CI**	1.3, 1.7	-0.3, 0.2	1.1, 1.5	-0.5, 0.0	1.2, 1.6	-0.2, 0.2	1.2, 1.7	-0.6, 0.1	1.3, 1.7	-0.2, 0.3
***p* value**		0.7792		0.1360		0.9648		0.0412		0.9698

*Only patients with a valid BL measurement and at least one valid follow-up assessment were included

†Within-group difference was tested using the Wilcoxon signed-rank test

BL: baseline; CI: confidence interval; EP, Endpoint; LOCF: last observation carried forward; PSP: Personal and Social Performance Scale; SD: standard deviation
